# One Decade of Online Patient Feedback: Longitudinal Analysis of Data From a German Physician Rating Website

**DOI:** 10.2196/24229

**Published:** 2021-07-26

**Authors:** Martin Emmert, Stuart McLennan

**Affiliations:** 1 Institute for Healthcare Management & Health Sciences University of Bayreuth Bayreuth Germany; 2 Institute of History and Ethics in Medicine Technical University of Munich Munich Germany; 3 Institute for Biomedical Ethics University of Basel Basel Switzerland

**Keywords:** physician rating websites, patient satisfaction, patient feedback, online ratings

## Abstract

**Background:**

Feedback from patients is an essential element of a patient-oriented health care system. Physician rating websites (PRWs) are a key way patients can provide feedback online. This study analyzes an entire decade of online ratings for all medical specialties on a German PRW.

**Objective:**

The aim of this study was to examine how ratings posted on a German PRW have developed over the past decade. In particular, it aimed to explore (1) the distribution of ratings according to time-related aspects (year, month, day of the week, and hour of the day) between 2010 and 2019, (2) the number of physicians with ratings, (3) the average number of ratings per physician, (4) the average rating, (5) whether differences exist between medical specialties, and (6) the characteristics of the patients rating physicians.

**Methods:**

All scaled-survey online ratings that were posted on the German PRW jameda between 2010 and 2019 were obtained.

**Results:**

In total, 1,906,146 ratings were posted on jameda between 2010 and 2019 for 127,921 physicians. The number of rated physicians increased constantly from 19,305 in 2010 to 82,511 in 2018. The average number of ratings per rated physicians increased from 1.65 (SD 1.56) in 2010 to 3.19 (SD 4.69) in 2019. Overall, 75.2% (1,432,624/1,906,146) of all ratings were in the best rating category of “very good,” and 5.7% (107,912/1,906,146) of the ratings were in the lowest category of “insufficient.” However, the mean of all ratings was 1.76 (SD 1.53) on the German school grade 6-point rating scale (1 being the best) with a relatively constant distribution over time. General practitioners, internists, and gynecologists received the highest number of ratings (343,242, 266,899, and 232,914, respectively). Male patients, those of higher age, and those covered by private health insurance gave significantly (*P*<.001) more favorable evaluations compared to their counterparts. Physicians with a lower number of ratings tended to receive ratings across the rating scale, while physicians with a higher number of ratings tended to have better ratings. Physicians with between 21 and 50 online ratings received the lowest ratings (mean 1.95, SD 0.84), while physicians with >100 ratings received the best ratings (mean 1.34, SD 0.47).

**Conclusions:**

This study is one of the most comprehensive analyses of PRW ratings to date. More than half of all German physicians have been rated on jameda each year since 2016, and the overall average number of ratings per rated physicians nearly doubled over the decade. Nevertheless, we could also observe a decline in the number of ratings over the last 2 years. Future studies should investigate the most recent development in the number of ratings on both other German and international PRWs as well as reasons for the heterogeneity in online ratings by medical specialty.

## Introduction

Feedback from patients is an essential element of a patient-oriented health care system [1]. Patients’ views and opinions on the care they have experienced can help health care organizations and professionals identify areas that need to be improved and can also help other patients with decision making when choosing where to receive health care [2]. Health care organizations and professionals can gather patient feedback in a variety of ways, including by conducting patient surveys, audits, interviews, focus groups, and deliberative events [3]. Patients have also always been able to actively share their views and opinions about the care they received with family and friends or with health care organizations and professionals via unsolicited comments or complaints. However, patients increasingly also have the ability to share their views and opinions on the internet and social media [4-7].

Physician rating websites (PRWs) are one of the key opportunities for patients to provide feedback online [4,7]. A systematic search of PRWs in 2018 identified 143 websites from 12 countries; however, the majority of websites were commercially operated in the United States and Germany [8]. Previous research involving PRW ratings in Germany and other countries has highlighted some common themes, including incomplete lists of physicians, a low number of physicians rated, a low number of ratings per physician that are overwhelmingly positive, and unstructured and different rating systems, which has raised concerns about the representativeness, validity, and usefulness of feedback on PRWs [7,9-30]. Medical associations have also often expressed strong opposition to PRWs, concerned that they will be used for doctorbashing or defamation [31,32]. Countries have different legal frameworks with regards to data protection, and previous research suggests that restrictive legal environments (eg, Switzerland) may be having an impact of the types of ratings on PRWs [28,29]. However, the legal basis for PRWs in Germany is reasonably liberal and well established. The Federal Court of Justice of Germany confirmed in 2014 the permissibility of ratings on the basis of the right to freedom of expression and that the anonymity of raters can only be lifted in exceptional cases [33,34]. Research also indicates that PRWs in Germany are having some success in influencing patient decision making and quality improvement [17,35].

However, most studies examining PRWs ratings have typically focused on a certain year (eg, [13,18,21]), a certain medical specialty (eg, [22,23,36-40]), certain cities or regions (eg, [14,26,41]), or with a (more or less) randomly selected sample of physicians or ratings (eg, [14,21,26,36,41]). There is therefore a need for a more comprehensive examination of PRW ratings, to reveal a more generalizable view of ratings and allow trends in rating habits to be identified. As far as we are aware, only 2 studies from the United States [13] and Canada [27] have presented such findings.

This study takes a different approach from most previous studies and analyzes an entire decade of online ratings for all medical specialties on the German PRW, jameda [14,21,26,42] (Please note that the data are not publicly available but may be provided from the provider of the website for research purposes upon request.). Jameda was founded in 2007 and since 2016, has been a wholly owned subsidiary of Burda Digital GmbH. The commercial website provides users with a categorized search function to find suitable physicians, the ability to make appointments with physicians online, the possibility to have video consultations with physicians, an encyclopedia with information from experts on health topics, and an opportunity to rate physicians on a predefined grading system or leave narrative comments. In Germany, a total of 25 PRWs have been identified [8]; however, previous research has indicated that jameda is the German PRW with the highest public awareness, usage, and number of ratings given [4,14,26].

The aim of this study was to examine how ratings posted on the German PRW jameda have developed over the past decade. In particular, it aimed to explore (1) the distribution of ratings according to time-related aspects (year, month, day of the week, and hour of the day) between 2010 and 2019, (2) the number of physicians with ratings, (3) the average number of ratings per physician, (4) the average rating, (5) whether differences exist between medical specialties, and (6) the characteristics of the patients rating physicians.

## Methods

### Overview

All scaled-survey online ratings that were posted on jameda between 2010 and 2019 were provided by jameda. Ratings on jameda are given according to the 6-point grading system used in German schools (1=very good, 2=good, 3=satisfactory, 4=fair, 5=deficient, and 6=insufficient) [24], in relation to 5 questions: (1) satisfaction with the treatment provided by the physician, (2) the physician’s explanation about the illness and treatment, (3) the relationship of trust with the physician, (4) the time the physician spent with the patient, and (5) friendliness of the physician. Additionally, a mean score (“overall performance”) is calculated based on the results for Q1 to Q5 [24]. The data also contained the physician’s year of birth and medical specialty, as well as the rating patient’s gender, age, and health insurance status.

### Statistical Analysis

Descriptive statistics included means and SDs for continuous variables as well as numbers and percentages for categorical variables. To analyze whether differences existed between 2 groups, the Mann-Whitney *U* test was used for continuous nonparametric variables, and the Kruskal-Wallis test was applied to determine differences between more than 2 groups. The Shapiro-Wilk test was used to examine the normality of the data distribution. Cohen *d* was calculated to measure the magnitude of the effect size by comparing the standardized difference between the means of 2 groups. All statistical analyses were conducted using SPSS version 22.0 (IBM Corp, Armonk, NY). Differences were considered to be significant if *P*<.05 and highly significant if *P*<.001.

## Results

### Distribution of Ratings and Mean Ratings

In total, 1,906,146 ratings were posted on jameda between 2010 and 2019 (see Table 1). The highest proportions of ratings were left in 2017 (293,744/1,906,146, 15.41%) and 2018 (292,721/1,906,146, 15.36%). In 2019, there was a decline in the number of ratings (232,739/1,906,146, 12.21%) in comparison with the previous years. Ratings were distributed throughout the months of the year relatively equally (minimum in December: 143,620/1,906,146, 7.53%; maximum in March: 173,865/1,906,146, 9.12%), but more variation was found by day of the week (minimum on Saturdays: 123,024/1,906,146, 6.45%; maximum on Tuesdays: 356,128/1,906,146, 18.68%) and by hour of the day (minimum during 3-4 am: 4659/1,906,146, 0.24%; maximum during 11-12 am: 152,606/1,906,146, 8.00%). Likewise, the mean ratings were relatively similar across years (minimum in 2019: mean 1.71, SD 1.52; maximum in 2013: mean 1.83, SD 1.56), months (minimum in January: mean 1.73, SD 1.49; maximum in August: mean 1.77, SD 1.54), and days (minimum on Sunday: mean 1.68, SD 1.45; maximum on Monday: mean 1.78, SD 1.54). However, more variation could be seen by hour of the day (minimum during 7-8 am: mean 1.67, SD 1.43; maximum during 2-3 am and 3-4 am: mean 2.05, SD 1.75 and mean 2.05, SD 1.72, respectively).

**Table 1:** Distribution of ratings (N=1,906,146) and mean ratings.

Timeframe			Ratings, n (%)		Mean rating, mean (SD)
**Year**					
	2010	31,908 (1.67)		1.73 (1.42)	
	2011	61,726 (3.23)		1.74 (1.44)	
	2012	98,041 (5.14)		1.77 (1.50)	
	2013	154,119 (8.08)		1.83 (1.56)	
	2014	219,319 (11.51)		1.81 (1.54)	
	2015	237,354 (12.45)		1.79 (1.54)	
	2016	284,475 (14.92)		1.71 (1.48)	
	2017	293,744 (15.41)		1.73 (1.52)	
	2018	292,721 (15.36)		1.78 (1.57)	
	2019	232,739 (12.21)		1.71 (1.52)	
**Month**					
	January	170,699 (9.00)		1.73 (1.49)	
	February	167,728 (8.80)		1.77 (1.53)	
	March	173,865 (9.11)		1.77 (1.53)	
	April	151,098 (7.93)		1.77 (1.53)	
	May	152,995 (8.02)		1.76 (1.53)	
	June	147,422 (7.73)		1.76 (1.53)	
	July	160,596 (8.43)		1.77 (1.53)	
	August	151,544 (7.95)		1.77 (1.54)	
	September	155,261 (8.15)		1.75 (1.52)	
	October	161,630 (8.48)		1.77 (1.53)	
	November	169,688 (8.90)		1.75 (1.52)	
	December	143,620 (7.53)		1.73 (1.51)	
**Day of the week**					
	Monday	342,025 (17.94)		1.78 (1.54)	
	Tuesday	356,128 (18.68)		1.78 (1.54)	
	Wednesday	329,457 (17.28)		1.75 (1.52)	
	Thursday	337,364 (17.70)		1.76 (1.53)	
	Friday	267,234 (14.02)		1.77 (1.54)	
	Saturday	123,024 (6.45)		1.74 (1.52)	
	Sunday	150,914 (7.91)		1.68 (1.45)	
**Hour of the day**					
	0-1	23,689 (1.24)		1.96 (1.68)	
	1-2	11,852 (0.62)		2.00 (1.71)	
	2-3	6686 (0.35)		2.05 (1.75)	
	3-4	4659 (0.24)		2.05 (1.72)	
	4-5	5151 (0.27)		1.98 (1.70)	
	5-6	9681 (0.51)		1.82 (1.57)	
	6-7	22,818 (1.20)		1.70 (1.47)	
	7-8	51,225 (2.69)		1.67 (1.43)	
	8-9	90,270 (4.74)		1.71 (1.47)	
	9-10	122,461 (6.42)		1.74 (1.50)	
	10-11	144,834 (7.60)		1.75 (1.51)	
	11-12	152,606 (8.01)		1.77 (1.53)	
	12-13	143,618 (7.53)		1.78 (1.54)	
	13-14	136,245 (7.15)		1.76 (1.53)	
	14-15	129,596 (6.80)		1.74 (1.50)	
	15-16	121,427 (6.37)		1.75 (1.52)	
	16-17	116,451 (6.11)		1.76 (1.53)	
	17-18	111,075 (5.83)		1.77 (1.54)	
	18-19	101,968 (5.35)		1.75 (1.53)	
	19-20	98,494 (5.17)		1.73 (1.52)	
	20-21	95,222 (5.00)		1.72 (1.51)	
	21-22	89,447 (4.69)		1.73 (1.51)	
	22-23	71,515 (3.75)		1.78 (1.54)	
	23-24	45,156 (2.37)		1.85 (1.60)	

### Number of Rated Physicians and Ratings Per Rated Physician

Between 2010 and 2019, a total of 127,921 physicians were rated on jameda (see Table 2). The number of rated physicians increased constantly from 19,305 in 2010 to 82,511 in 2018. In 2019, the number of rated physicians decreased to 73,071 rated physicians. The number of ratings that rated physicians received demonstrated an increasing trend. In 2010, 66.94% (12,923/19,305) of all rated physicians were rated only once, 30.88% (5961/19,305) were rated 2-5 times, 1.71% (330/19,305) were rated 6-10 times, and 0.47% (91/19,305) were rated 11-50 times. In 2019, 40.84% (29,843/73,071) of all rated physicians were rated only once, 46.89% (34,262/73,071) were rated 2-5 times, 8.21% (5998/73,071) were rated 6-10 times, 3.93% (2875/73,071) were rated 11-50 times, and 0.13% (93/73,071) were rated more than 50 times. Over the entire decade, 11.43% (14,625/127,921) of all rated physicians were rated once, and 4.23% (5413/127,921) were rated more than 50 times. Please note that the overall numbers cannot be summed up here. For example, one physician received 1 rating in 2010, 3 ratings in 2011, 5 ratings in 2013, 1 rating in 2015, 11 ratings in 2015, 23 ratings in 2017, and 19 ratings in 2019. In sum, this physician was rated 63 times and would be assigned to the category “≥51 Ratings.” Similarly, the overall average number of ratings per rated physician increased from 1.65 (SD 1.56) in 2010 to 3.19 (SD 4.69) in 2019. Comparing the number of ratings and rated physicians with the total number of physicians in the German outpatient sector [43], in 2010, 13.64% (19,305/141,461) of all physicians had been rated on jameda, 21.93% (31,335/142,855) in 2011, 29.22% (42,089/144,058) in 2012, 36.36% (53,065/145,933) in 2013, 42.71% (63,182/147,948) in 2014, 45.56% (68,392/150,106) in 2015, 50.51% (76,773/151,989) in 2016, 51.69% (79,799/154,369) in 2017, and 52.46% (82,511/157,288) in 2018 (see also Multimedia Appendix 1). Thus, more than half of all German physicians have been rated online on jameda each year in Germany since 2016.

**Table 2:** Overall ratings on jameda between 2010 and 2019.

Ratings		Year											Overall (n=127,921)
		2010 (n=19,305)	2011 (n=31,336)	2012 (n=42,089)	2013 (n=53,065)	2014 (n=63,182)	2015 (n=68,392)	2016 (n=76,773)	2017 (n=79,799)	2018 (n=82,511)	2019 (n=73,071)		
**Overall number and percentage of rated physicians, n (%)**													
	1 rating	12,923 (66.94)	18,256 (58.26)	21,133 (50.21)	22,177 (41.79)	22,229 (35.18)	24,512 (35.84)	25,859 (33.68)	26,810 (33.60)	28,971 (35.11)	29,843 (40.84)	14,625 (11.43)	
	2-5 ratings	5961 (30.88)	11,877 (37.90)	18,389 (43.69)	25,321 (47.71)	31,422 (49.73)	33,751 (49.35)	38,263 (49.84)	39,808 (49.89)	40,602 (49.21)	34,262 (46.89)	31,507 (24.63)	
	6-10 ratings	330 (1.71)	933 (2.98)	1936 (4.60)	4085 (7.70)	6755 (10.69)	7061 (10.32)	8710 (11.35)	9099 (11.40)	9007 (10.92)	5998 (8.21)	26,285 (20.55)	
	11-50 ratings	91 (0.47)	259 (0.83)	604 (1.44)	1424 (2.68)	2683 (4.25)	2954 (4.32)	3787 (4.93)	3933 (4.93)	3801 (4.61)	2875 (3.93)	50,091 (39.16)	
	≥51 ratings	0 (0.00)	11 (0.00)	27 (0.01)	58 (0.11)	93 (0.15)	114 (0.12)	154 (0.20)	149 (0.19)	130 (0.16)	93 (0.13)	5413 (4.23)	
Percentage of rated physicians, % (N)		13.64 (141,461)	21.93 (142,855)	29.22 (144,058)	36.36 (145,933)	42.71 (147,948)	45.56 (150,106)	50.51 (151,989)	51.69 (154,369)	52.46 (157,288)	N/A^a^	–^b^	
**Number of ratings per rated physician**													
	Mean (SD)	1.65 (1.56)	1.97 (2.51)	2.33 (3.22)	2.90 (4.05)	3.47 (4.84)	3.47 (4.95)	3.71 (5.43)	3.68 (5.09)	3.55 (4.92)	3.19 (4.69)	14.90 (24.04)	
	Maximum	39	137	151	149	165	154	197	143	215	148	943	

^a^N/A: not available.

^b^Not applicable.

### Rating Evaluations

Of the 1,906,146 ratings posted between 2010 and 2019, 75.16% (1,432,624/1,906,146) of all ratings were in the best rating category of “very good,” and 5.66% (107,912/1,906,146) of the ratings were in the lowest category of “insufficient” (see Table 3). Furthermore, the percentage of ratings on both ends of the rating scale increased over time, from 71.95% (2010) to 78.17% (2019) for very positive ratings and from 3.91% (2010) to 6.12% (2019) for very negative ratings. However, the overall average rating remained relatively constant. The average rating was 1.73 (SD 1.42) in 2010 and 1.71 (SD 1.52) in 2019, with an overall average of 1.76 (SD 1.53).

**Figure 1:**
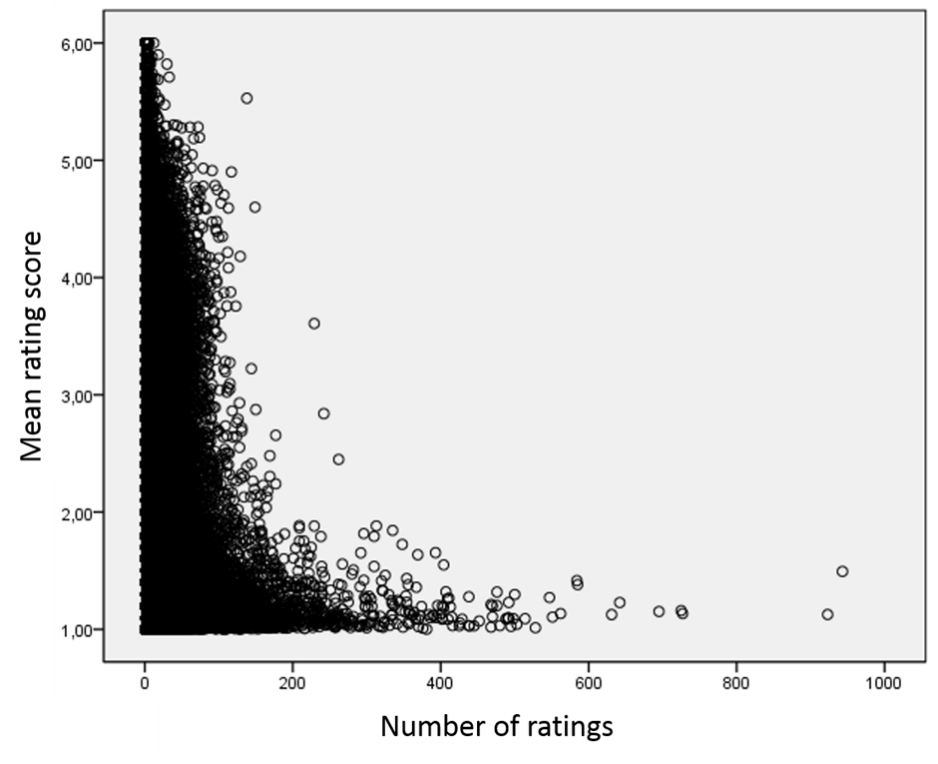
Scatterplot (bivariate) of the number of ratings per physician with the mean overall performance for rated physicians.

With regards to the correlation between the average rating of a rated physician and the number of ratings per physician, physicians with a lower number of ratings tended to receive ratings across the rating scale, while physicians with a higher number of ratings tended to have better ratings (see Figure 1). Physicians with a single rating had a mean rating of 1.58 (SD 1.28). Afterwards, mean ratings get worse with increasing number of ratings. Physicians with between 21 and 50 online ratings received the worst ratings (mean 1.95, SD 0.84). Mean ratings then improve, with physicians having 51-100 ratings receiving a mean rating of 1.79 (SD 0.86) and physicians with more than 100 ratings receiving the best ratings (mean 1.34, SD 0.47; see Table 4).

**Table 3:** Overall rating evaluations on jameda between 2010 and 2019.

Overall rating evaluation		Year											Overall (n=1,906,146)
		2010 (n=31,908)	2011 (n=61,726)	2012 (n=98,041)	2013 (n=154,119)	2014 (n=219,319)	2015 (n=237,354)	2016 (n=284,475)	2017 (n=293,744)	2018 (n=292,721)	2019 (n=232,729)		
**Rating based on the 6-point grading system, n (%)**													
	1=very good	22,957 (71.95)	44,952 (72.83)	72,066 (73.51)	111,043 (72.05)	160,263 (73.07)	175,416 (73.90)	217,533 (76.47)	224,527 (76.44)	221,951 (75.82)	181,916 (78.17)	1,432,624 (75.16)	
	2=good	3406 (10.67)	5783 (9.37)	7889 (8.05)	12,113 (7.86)	16,651 (7.59)	17,328 (7.30)	19,383 (6.81)	18,489 (6.29)	17,205 (5.88)	12,203 (5.24)	130,450 (6.84)	
	3=satisfactory	1036 (3.25)	2007 (3.25)	2920 (2.98)	4766 (3.09)	6200 (2.83)	6321 (2.66)	6655 (2.34)	6848 (2.33)	6665 (2.28)	4491 (1.93)	47,909 (2.51)	
	4=fair	1312 (4.11)	2635 (4.27)	4082 (4.16)	6631 (4.30)	9073 (4.14)	9444 (3.98)	9545 (3.36)	9678 (3.29)	10,021 (3.42)	7056 (3.03)	69,477 (3.64)	
	5=deficient	1948 (6.11)	3910 (6.33)	6233 (6.36)	10,694 (6.94)	15,121 (6.89)	15,658 (6.60)	16,493 (5.80)	17,339 (5.90)	17,537 (5.99)	12,841 (5.52)	117,774 (6.18)	
	6=insufficient	1249 (3.91)	2439 (3.95)	4851 (4.95)	8872 (5.76)	12,011 (5.48)	13,187 (5.56)	14,866 (5.23)	16,863 (5.74)	19,342 (6.61)	14,232 (6.12)	107,912 (5.66)	
Mean (SD)		1.73 (1.42)	1.74 (1.44)	1.77 (1.50)	1.83 (1.56)	1.81 (1.54)	1.79 (1.54)	1.71 (1.48)	1.73 (1.52)	1.78 (1.57)	1.71 (1.52)	1.76 (1.53)	

**Table 4:** Online rating results by the number of ratings per physician.

Number of ratings per physician	Average rating, mean (SD)	Rating based on the 6-point grading system^a^, n (%)					
		1	2	3	4	5	6
1 (n=14,625)	1.58 (1.28)	11,741 (80.28)	1023 (6.99)	302 (2.06)	394 (2.69)	645 (4.41)	520 (3.56)
2-5 (n=31,505)	1.67 (0.96)	19,733 (62.63)	6204 (19.69)	3477 (11.04)	1395 (4.43)	471 (1.50)	225 (0.71)
6-10 (n=26,258)	1.76 (0.81)	12,505 (47.62)	9459 (36.02)	3096 (11.79)	961 (3.66)	214 (0.81)	23 (0.09)
11-20 (n=29,049)	1.86 (0.78)	12,243 (42.15)	11,188 (38.51)	4289 (14.76)	1162 (4.00)	159 (0.55)	8 (0.02)
21-50 (n=20,658)	1.95 (0.84)	8044 (38.94)	7833 (37.92)	3408 (16.50)	1195 (5.78)	176 (0.85)	2 (0.00)
51-100 (n=3933)	1.79 (0.86)	2084 (52.99)	1122 (28.53)	446 (11.34)	246 (6.25)	35 (0.89)	0 (0.00)
>100 (n=1445)	1.34 (0.47)	1181 (81.73)	220 (15.22)	28 (1.94)	10 (0.69)	5 (0.35)	1 (0.07)
Total (n=127,473)	1.77 (0.92)	67,531 (52.98)	37,049 (29.06)	15,046 (11.80)	5363 (4.21)	1705 (1.34)	779 (0.61)

^a^1=very good, 2=good, 3=satisfactory, 4=fair, 5=deficient, and 6=insufficient.

### Ratings by Medical Specialty

Between 2010 and 2019, general practitioners (343,242), internists (266,899), gynecologists (232,914), and orthopedists (229,481) received the highest number of ratings, while pediatricians (87,330), ophthalmologists (79,699), and urologists (63,703) received the lowest number of ratings (see Table 5). However, according to the relative distribution of ratings, the most frequently rated medical specialties in 2018 were orthopedists (6160/7302, 84.36%); oral maxillofacial surgeons (1017/1257, 80.91%); ear, nose, and throat (ENT) specialists (3559/4479, 79.46%); and dermatologists (3562/4632, 76.90%). In contrast, the least frequently rated medical specialties were radiologists (863/4078, 21.16%) and anesthesiologists (601/4247, 14.15%; see Multimedia Appendix 2). Among the 10 most frequently rated medical specialties, the best rated medical specialties were urologists (mean 1.50, SD 1.29), general practitioners (mean 1.64, SD 1.40), and internists (mean 1.68, SD 1.45). The lowest ratings were given to pediatricians (mean 1.92, SD 1.62), ophthalmologists (mean 2.06, SD 1.74), and dermatologists (mean 2.11, SD 1.77).

**Table 5:** Ratings by medical specialty.

Medical specialty		Year												Overall (n=127,921 rated physicians; n=1,906,146 ratings)
		2010 (n=19,305 rated physicians; n=31,908 ratings)		2011 (n=31,336 rated physicians; n=61,726 ratings)	2012 (n=42,089 rated physicians; n=98,041 ratings)	2013 (n=53,065 rated physicians; n=154,119 ratings)	2014 (n=63,182 rated physicians; n=219,319 ratings)	2015 (n=68,392 rated physicians; n=237,354 ratings)	2016 (n=76,773 rated physicians; n=284,475 ratings)	2017 (n=79,799 rated physicians; n=293,744 ratings)	2018 (n=82,551 rated physicians; n=292,721 ratings)	2019 (n=73,071 rated physicians; n=232,729 ratings)		
**General practitioner**														
	Rated physicians, n (%)		4891 (25.34)	8161 (26.04)	10,533 (25.03)	13,077 (24.64)	15,767 (24.95)	17,016 (24.88)	19,289 (25.12)	19,586 (24.54)	19,967 (24.19)	16,818 (23.02)	33,414 (26.12)	
	Number of ratings		7241 (22.69)	13,737 (22.25)	19,210 (19.59)	27,952 (18.14)	39,914 (18.20)	42,188 (17.77)	51,504 (18.10)	51,725 (17.61)	51,682 (17.66)	38,089 (16.37)	343,242 (18.01)	
	Mean rating (SD)^a^, n (%)		1.55 (1.21)	1.53 (1.21)	1.54 (1.24)	1.60 (1.33)	1.61 (1.35)	1.66 (1.40)	1.59 (1.35)	1.65 (1.43)	1.73 (1.52)	1.71 (1.51)	1.64 (1.40)	
**Internist**														
	Rated physicians, n (%)		3230 (16.73)	5286 (16.87)	6897 (16.39)	8779 (16.54)	10,635 (16.83)	11,511 (16.83)	13,374 (17.42)	13,849 (17.35)	14,634 (17.73)	13,306 (18.21)	23,734 (18.55)	
	Number of ratings, n (%)		5132 (16.08)	9381 (15.20)	13,697 (13.97)	20,853 (13.53)	29,728 (13.55)	31,611 (13.32)	39,619 (13.93)	40,616 (13.83)	41,642 (14.23)	34,620 (14.88)	266,899 (14.00)	
	Mean rating (SD)^a^		1.59 (1.27)	1.62 (1.32)	1.62 (1.36)	1.70 (1.44)	1.68 (1.43)	1.70 (1.46)	1.63 (1.40)	1.68 (1.47)	1.73 (1.53)	1.68 (1.49)	1.68 (1.45)	
**Gynecologist**														
	Rated physicians, n (%)		2157 (11.17)	3568 (11.39)	5084 (12.08)	6291 (11.86)	7163 (11.34)	7602 (11.12)	8165 (10.64)	8445 (10.58)	8653 (10.48)	7650 (10.47)	11,598 (9.07)	
	Number of ratings, n (%)		3901 (12.23)	7800 (12.64)	13,987 (14.27)	21,880 (14.20)	28,672 (13.07)	29,795 (12.55)	33,862 (11.90)	34,530 (11.76)	33,562 (11.47)	24,925 (10.71)	232,914 (12.22)	
	Mean rating (SD)^a^		1.66 (1.36)	1.64 (1.33)	1.69 (1.41)	1.79 (1.49)	1.79 (1.50)	1.80 (1.51)	1.74 (1.49)	1.73 (1.48)	1.76 (1.52)	1.69 (1.47)	1.75 (1.48)	
**Orthopedist**														
	Rated physicians, n (%)		1662 (8.61)	2548 (8.13)	3333 (7.92)	4007 (7.55)	4629 (7.33)	5051 (7.39)	5579 (7.27)	5907 (7.40)	6160 (7.46)	5894 (8.07)	8022 (6.27)	
	Number of ratings, n (%)		3412 (10.69)	6836 (11.07)	11,020 (11.24)	17,805 (11.55)	25,714 (11.72)	28,876 (12.17)	34,242 (12.04)	36,416 (12.40)	35,564 (12.15)	29,596 (12.72)	229,481 (12.04)	
	Mean rating (SD)^a^		2.08 (1.67)	2.06 (1.67)	2.12 (1.75)	2.15 (1.78)	2.05 (1.72)	1.93 (1.65)	1.82 (1.58)	1.80 (1.57)	1.82 (1.60)	1.70 (1.52)	1.89 (1.63)	
**Dermatologist (including venereologist)**														
	Rated physicians, n (%)		855 (4.43)	1354 (4.32)	1947 (4.63)	2467 (4.65)	2811 (4.45)	3003 (4.39)	3229 (4.21)	3415 (4.28)	3562 (4.31)	3232 (4.42)	4517 (3.53)	
	Number of ratings, n (%)		1563 (4.90)	3199 (5.18)	5811 (5.93)	10,461 (6.79)	14,991 (6.84)	15,380 (6.48)	17,513 (6.16)	17,619 (6.00)	17,861 (6.10)	13,355 (5.74)	117,753 (6.18)	
	Mean rating (SD)^a^		2.06 (1.64)	2.18 (1.71)	2.35 (1.85)	2.28 (1.82)	2.25 (1.82)	2.16 (1.77)	2.05 (1.73)	2.04 (1.74)	2.04 (1.75)	1.94 (1.71)	2.11 (1.77)	
**ENT^b^ specialist, otorhinolaryngologist**														
	Rated physicians, n (%)		835 (4.33)	1388 (4.43)	1876 (4.46)	2425 (4.57)	2828 (4.45)	3094 (4.52)	3345 (4.36)	3443 (4.31)	3559 (4.31)	3233 (4.42)	4709 (3.68)	
	Number of ratings, n (%)		1455 (4.56)	3018 (4.89)	5081 (5.18)	9013 (5.85)	13,494 (6.15)	14,626 (6.16)	17,107 (6.01)	16,914 (5.76)	16,118 (5.51)	13,077 (5.62)	109,903 (5.77)	
	Mean rating (SD)^a^		1.81 (1.50)	1.77 (1.46)	1.76 (1.50)	1.83 (1.57)	1.75 (1.51)	1.74 (1.52)	1.64 (1.43)	1.67 (1.47)	1.75 (1.56)	1.71 (1.53)	1.72 (1.51)	
**General surgery**														
	Rated physicians, n (%)		601 (3.11)	1027 (3.28)	1397 (3.32)	1836 (3.46)	2150 (3.40)	2463 (3.60)	2791 (3.64)	3054 (3.83)	3154 (3.82)	2859 (3.91)	4343 (3.40)	
	Number of ratings, n (%)		1061 (3.33)	2298 (3.72)	3661 (3.73)	6103 (3.96)	9084 (4.14)	10,908 (4.60)	13,240 (4.65)	14,678 (5.00)	14,162 (4.84)	12,272 (5.27)	87,467 (4.59)	
	Mean rating (SD)^a^		1.80 (1.49)	1.84 (1.57)	1.83 (1.59)	1.83 (1.59)	1.84 (1.61)	1.83 (1.62)	1.79 (1.60)	1.81 (1.63)	1.85 (1.67)	1.78 (1.62)	1.82 (1.62)	
**Pediatrician**														
	Rated physicians, n (%)		976 (5.06)	1570 (5.01)	2321 (5.51)	2996 (5.65)	3574 (5.66)	3891 (5.69)	4230 (5.51)	4315 (5.41)	4364 (5.29)	3620 (4.95)	6555 (5.12)	
	Number of ratings, n (%)		1529 (4.81)	2795 (4.53)	4941 (5.04)	7831 (5.08)	11,059 (5.04)	11,550 (4.87)	13,004 (4.57)	13,295 (4.53)	12,894 (4.40)	8432 (3.62)	87,330 (4.58)	
	Mean rating (SD)^a^		1.68 (1.35)	1.70 (1.38)	1.76 (1.46)	1.88 (1.57)	1.94 (1.61)	1.94 (1.61)	1.90 (1.60)	1.93 (1.64)	2.03 (1.72)	2.01 (1.73)	1.92 (1.62)	
**Ophthalmologist**														
	Rated physicians, n (%)		722 (3.74)	1225 (3.91)	1772 (4.21)	2366 (4.46)	2922 (4.62)	3131 (4.58)	3528 (4.60)	3809 (4.77)	3916 (4.74)	3520 (4.82)	5935 (4.64)	
	Number of ratings, n (%)		1085 (3.40)	2079 (3.37)	3570 (3.64)	6173 (4.01)	9154 (4.17)	9754 (4.11)	11,899 (4.18)	12,816 (4.36)	12,887 (4.40)	10,282 (4.41)	79,699 (4.18)	
	Mean rating (SD)^a^		2.07 (1.63)	2.09 (1.67)	2.26 (1.81)	2.20 (1.79)	2.15 (1.78)	2.11 (1.76)	1.98 (1.69)	1.97 (1.69)	2.05 (1.77)	1.96 (1.71)	2.06 (1.74)	
**Urologist**														
	Rated physicians, n (%)		536 (2.78)	830 (2.65)	1221 (2.90)	1511 (2.85)	1820 (2.88)	1914 (2.80)	2139 (2.79)	2301 (2.88)	2415 (2.93)	2140 (2.93)	3329 (2.60)	
	Number of ratings, n (%)		845 (2.65)	1639 (2.66)	3221 (3.29)	5141 (3.34)	7207 (3.29)	7753 (3.27)	9556 (3.36)	10,264 (3.49)	9612 (3.28)	8465 (3.64)	63,703 (3.34)	
	Mean rating (SD)^a^		1.82 (1.54)	1.66 (1.37)	1.57 (1.33)	1.64 (1.41)	1.54 (1.30)	1.50 (1.28)	1.43 (1.20)	1.47 (1.26)	1.49 (1.29)	1.45 (1.25)	1.50 (1.29)	
**Others**														
	Rated physicians, n (%)		2840 (14.71)	4379 (13.97)	5708 (13.56)	7310 (13.78)	8883 (14.06)	9716 (14.21)	11,104 (14.46)	11,675 (14.63)	12,127 (14.69)	10,799 (14.78)	21,765 (17.01)	
	Number of ratings, n (%)		4684 (14.68)	8944 (14.49)	13,842 (14.12)	20,907 (13.57)	30,302 (13.82)	34,913 (14.71)	42,929 (15.09)	44,871 (15.28)	46,737 (15.97)	39,626 (17.03)	287,755 (15.10)	
	Mean rating (SD)^a^		1.78 (1.48)	1.77 (1.49)	1.68 (1.45)	1.76 (1.54)	1.70 (1.49)	1.65 (1.45)	1.59 (1.40)	1.62 (1.43)	1.66 (1.49)	1.57 (1.41)	1.65 (1.45)	

^a^On a 6-point scale: 1=very good, 2=good, 3=satisfactory, 4=fair, 5=deficient, and 6=insufficient.

^b^ENT: ear, nose, throat.

### Characteristics of Raters

The rating patients were mostly female (56.8%), between 30 and 50 years old (42.6%), and covered by Statutory Health Insurance (81.0%; see Table 6). However, there were some significant differences between genders, age groups, and health insurance status. Male patients gave significantly more favorable ratings than female patients (mean rating 1.61, SD 1.32 vs. mean 1.77, SD 1.48; *P*<.001). Older patients also gave significantly better ratings than younger patients (*P*<.001). For example, patients aged 51 years or older left a mean rating of 1.52 (SD 1.22), whereas patients aged 29 years or younger left a mean rating of 1.93 (SD 1.59). Finally, patients covered by private health insurance (mean rating 1.43, SD 1.11) gave significantly more favorable evaluations than did patients covered by statutory health insurance (mean rating 1.75, SD 1.47; *P*<.001). Nevertheless, effect sizes were small for all groups, varying between 0.114 and 0.289.

**Table 6:** Characteristics of raters.

Characteristic			Number of respondents, n (%)		Rating evaluation, mean (SD)		*P* value		Cohen *d*
**Gender (n=1,107,092)**									
	Male	478,592 (43.23)		1.61 (1.32)		<.001^a^		0.114	
	Female	628,500 (56.77)		1.77 (1.48)					
**Age (years; n=1,063,523)**									
	≤29	164,807 (15.50)		1.93 (1.59)		<.001^b^		0.117^c^; 0.289^d^; 0.171^e^	
	30-50	452,774 (42.57)		1.75 (1.46)					
	≥51	445,942 (41.93)		1.52 (1.22)					
**Health insurance (n=981,635)**									
	Statutory health insurance	795,107 (81.00)		1.75 (1.47)		<.001^a^		0.245	
	Private health insurance	186,528 (19.00)		1.43 (1.11)					

^a^Mann-Whitney *U* test.

^b^Kruskal-Wallis test.

^c^≤29 years vs 30-50 years.

^d^≤29 years vs ≥51 or years.

^e^30-50 years vs ≥51 years.

## Discussion

This study is one of the most comprehensive analyses of PRW ratings conducted to date and has resulted in a number of key findings: (1) just under 2 million ratings were posted on jameda between 2010 and 2019; (2) a total of 127,921 physicians were rated; (3) the overall average number of ratings per rated physicians nearly doubled; (4) three-quarters of all ratings were in the best rating category of “very good,” and the overall average rating remained relatively constant; (5) general practitioners, internists, gynecologists, and orthopedists were the most frequently rated medical specialties; and (6) the rating patients were mostly female, between 30 and 50 years old, and covered by Statutory Health Insurance.

The findings of this study confirm previous research in Germany that indicated that patient ratings show an increasing trend over the past decade [26]. For example, the percentage of all German physicians that had been rated on jameda increased constantly over time from 13.65% (19,305/141,461) in 2010 to 52.46% (82,511/157,288) in 2018. McLennan et al [26] also previously reported that the proportion of physicians from a sample of 298 randomly selected physicians from Hamburg and Thuringia that had been rated at least once had increased between 2010 (range 3.3%-27.8%) and 2014 (range 16.4%-83.2%). Similarly, the average number of ratings per physician also increased between 2010 (range 1.1-3.1) and 2014 (range 1.2-7.5). However, this study only used a small sample from 2 regions in Germany. Overall, there is little international evidence showing the exact development of online ratings over time, which makes it challenging to compare our numbers with those from other similar studies. To the best of our knowledge, more recent studies providing detailed information on a yearly basis are limited. However, 2 studies from the United States [13] and Canada [27] have presented similar findings. First, in 2012, Gao and colleagues [13] showed an increase in the number of rated physicians on RateMDs in the United States from 2475 in 2005 to 112,024 in 2010. Second, Liu and colleagues [27] analyzed a dataset from RateMDs, which included all physicians in Canada in 2018 and showed an increase in the number of ratings for physicians in Canada from 138 in 2005 to 640,603 in 2013. Nevertheless, it should be noted that this study found a plateau in the total number of ratings between 2017 (293,744) and 2018 (292,721). In 2019, a decrease of around 20% in the total number of ratings was seen in comparison with the previous 2 years. In recent years, jameda has implemented and promoted new features on its website (eg, making appointments, video consultations). This has possibly led to lower marketing efforts for collecting online reviews and may also lead to differences from PRWs not offering these addition services. Future studies should investigate whether this latest development can also be observed for other PRWs in Germany and other countries.

This study only provides information regarding jameda. Previous research has demonstrated much lower numbers of both ratings and rated physicians on other German PRWs [4,26]. For example, McLennan and colleagues [26] reported that between 16.4% and 71.1% (mean 41.4%) of physicians were rated on German PRWs overall, compared with 83.2% on jameda. Another study also showed a higher percentage of rated physicians on jameda (90.2%) compared with other relevant German PRWs (32.4% to 61.2%) [4]. Differences in the number of ratings between PRWs can also be shown in the international setting. For example, Trehan and colleagues [44] analyzed online ratings for 250 hand surgeons from the American Society for Surgery of the Hand member directory from 3 PRWs in the United States (HealthGrades, Vitals, RateMDs). Large differences were reported regarding the average number of ratings (13.4, 8.3, and 1.9, respectively) [44]. Further research is required to confirm that this increase in ratings is also true for other PRWs as well.

Furthermore, the percentages of ratings on both ends of the rating scale have increased. This may suggest that a “bimodal” trend in ratings is emerging on jameda, similar to that seen with the rating of products on websites like Amazon where “amateur” reviewers usually only leave a review because they either love or hate a product [45]. It would be helpful if future research examines if this trend continues and can be found on other PRWs, particularly as this trend is usually not seen on PRWs [26], despite qualitative research in Germany finding that a very positive or very negative experience in the health care relationship is a crucial precondition for patients to be willing to rate a physician [46].

Seven years after the first study on online patient ratings on jameda [18], general practitioners, internists, and gynecologists still receive the highest number of ratings in absolute terms. This does not seem surprising due to the high number of physicians in those medical specialty areas in Germany. Similar to previous research [18], it could also be shown that urologists, general practitioners, and internists were likely to receive more favorable ratings on jameda. In contrast, ophthalmologists and dermatologists are still likely to receive far less favorable ratings. This is also in line with the comprehensive analysis by Liu and colleagues [27] from Canada. Previous research findings have also reported that generalists are more likely to have better online ratings than specialists [10,13]. Qualitative research conducted in Germany by McLennan et al [46] found that factors concerning the physician-patient relationship to be some of the most important influencing people’s willingness to rate their physician on PRWs. It is likely that differences in patients’ relationships with physicians in various specialties (eg, duration and frequency of contact and the resulting level of trust) is a key factor for this heterogeneity.

The analysis of such a large number of ratings has also provided a more detailed picture of the association between the number of ratings a physician has and their overall evaluation. Although physicians with only 1 rating tended to have very good ratings (81% of all ratings were in the best rating category), this might potentially be explained, at least in part, by “fake ratings” left by physicians themselves or people connected to the physician. Regardless, it certainly calls into question whether results based on a single rating are meaningful at all [7]. Afterwards, more critical rating results were found. In line with previous studies from Germany [18] and the United States [37], the total performance range was found for physicians with a lower number of ratings. This possibly represents a more realistic picture of patient feedback because the percentage of ratings in the very best rating category declined constantly, and it is also likely that those physicians are not using PRWs as a marketing measure to collect a very high number or ratings [18]. However, in contrast to previous research, physicians who received a higher number of ratings were shown to have better ratings. When there were more than 51 ratings, ratings started to improve again, and physicians with more than 100 ratings received by far the most favorable ratings. It is likely that physician with more than 100 ratings are aware of PRWs and are using them as a marketing tool, potentially specifically asking satisfied patients to leave a (positive) rating on a PRW. However, it is possible that these physicians are simply providing outstanding quality of care, leading to the very favorable ratings on PRWs and, subsequently, more patients choosing to use this physician [18]. Future research should examine which assumption is true [18].

In 2019, Pike et al [37] reported a U-shaped relationship between the number of ratings and the overall rating from the Healthgrades website. A negative relationship between the number of ratings and the overall rating could be seen until physicians achieved 21 ratings; thereafter, a positive relationship was seen. It should be noted that, in contrast to jameda, a lower score on Healthgrades means a worse rating (1=poor; 5=excellent). Although regression analysis on the jameda data did not find a satisfying fit, the study provides further broad-scale evidence on the relationship between the number of ratings and the overall evaluation as discussed earlier in this manuscript.

### Limitations

The key limitation of this study is that it analyzed online ratings from only a single German PRW, jameda. Although jameda has shown to be the most frequently used German PRW, there are a total of 25 PRWs in Germany [8], and it is unclear how generalizable the results are to other German PRWs or to other countries. In Germany, it would be particularly helpful for future longitudinal research to examine trends in ratings on PRWs run by public health insurers, as previous research has indicated that these PRWs have been able to quickly establish themselves as some of the most used German PRWs alongside jameda [26]. Another limitation of the study is that it only analyzed publicly available ratings; it is not known how many additional ratings jameda received but did not publish or what efforts jameda made to check whether published ratings are genuine and not fake. Indeed, jameda has often been criticized with regards to the number of fake reviews and its business model that offers physicians paid premium profiles. Recent research has raised concerns that online patient feedback is being inappropriately manipulated by many PRWs and that business models that make PRWs reliant on paying physicians may create financial incentives to suppress negative feedback [47]. Although further work is needed on criteria for determining which feedback is published [47], it is also important to have a comprehensive understanding of the ratings that are being viewed by the public on PRWs.

### Conclusion

In conclusion, it can be stated that online ratings have been increasing tremendously over the past decade and seem to have become an essential element for patients to leave feedback on the care they receive. More than half of all physicians have been rated online on jameda each year in Germany since 2016. Indeed, with patients increasingly using the internet in relation to their health care [48], it is likely that online patient feedback will become even more important in the future. With online patient feedback mostly positive, physicians do not have to fear online ratings in general; the commonly expressed concerns regarding PRWs being used for “doctorbashing” or defamation [31] or as “platforms for denunciation” [32] have not proven true. Furthermore, less favorable patient ratings often address important elements of a patient-oriented health care system [1] and can help organizations and professionals identify areas that need to be improved [21].

## Appendix

Multimedia Appendix 1 The number and percentage of rated physicians in Germany.

Multimedia Appendix 2 Number and distribution of ratings according to the medical specialty (2018).

## Abbreviations

ENT: ear, nose, and throat
PRW: physician rating website
