# Supplementary figures and images for: One Decade of Online Patient Feedback: Longitudinal Analysis of Data From a German Physician Rating Website
Source: J Med Internet Res. 2021 Jul 26;23(7):e24229. doi: 10.2196/24229 (PMC8367114; doi:10.2196/24229)

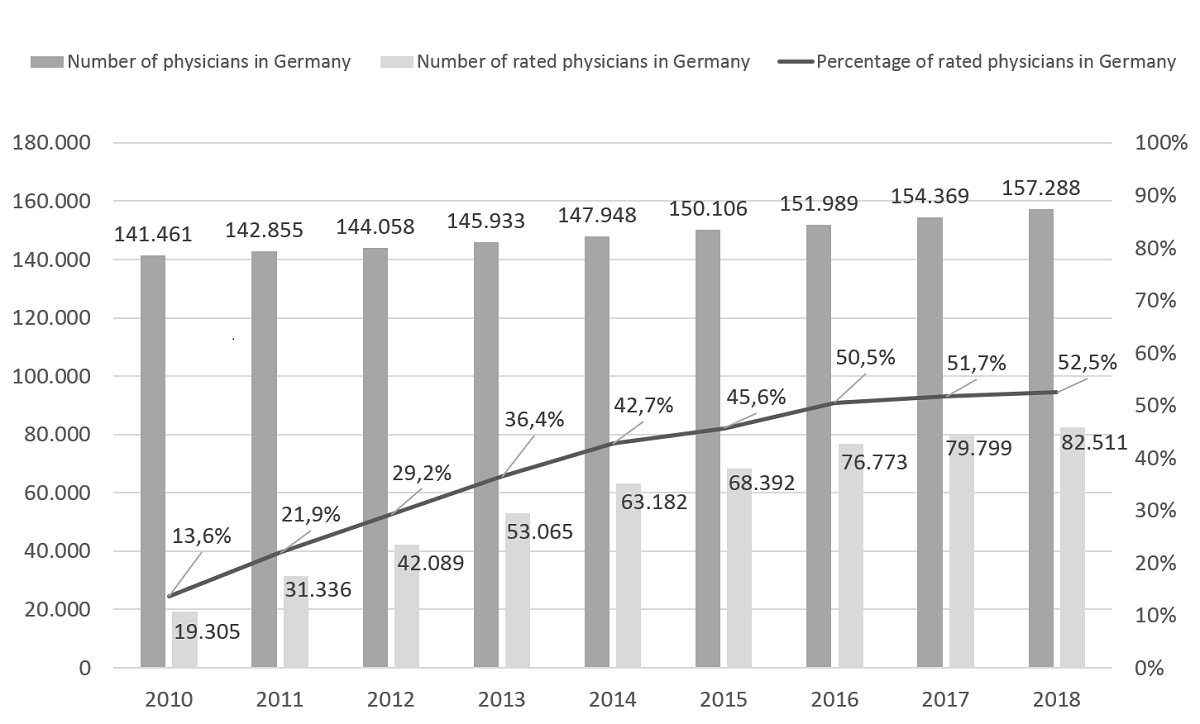

Supplement: Multimedia Appendix 1 [file jmir_v23i7e24229_app1.png]
