# Supplementary material for: One Decade of Online Patient Feedback: Longitudinal Analysis of Data From a German Physician Rating Website
Source: J Med Internet Res. 2021 Jul 26;23(7):e24229. doi: 10.2196/24229 (PMC8367114; doi:10.2196/24229)
Supplement: Multimedia Appendix 2 [file jmir_v23i7e24229_app2.docx]

Online appendix 2. Number and distribution of ratings according to the medical specialty (2018)

|  | Medical specialty | Number and distribution of ratings (2018) | | |
| --- | --- | --- | --- | --- |
|  |  | Rated physicians in  absolute terms  [N (%)] | Number of physicians in Germany^a^ | Rated physicians in relative terms (in %) |
| 1 | Orthopedist | 6,160 (7.5) | 7,302 | 84.4 |
| 2 | Oral maxillo-facial surgeon | 1,017 (1.2) | 1,257 | 80.9 |
| 3 | ENT specialist, otorhinolaryngologist | 3,559 (4.3) | 4,479 | 79.5 |
| 4 | Dermatologist (incl. venereologist) | 3,562 (4.3) | 4,632 | 76.9 |
| 5 | Urologist | 2,415 (2.9) | 3,198 | 75.5 |
| 6 | Gynecologist | 8,653 (10.5) | 11,753 | 73.6 |
| 7 | Medical practitioner without specialization | 2,226 (2.7) | 3,179 | 70.0 |
| 8 | Ophthalmologist | 3,916 (4.7) | 6,294 | 62.2 |
| 9 | General surgery | 3,154 (3.8) | 5,038 | 62.6 |
| 10 | Neurologist/Psychiatrist | 3,821 (4.6) | 6,527 | 58.5 |
| 11 | Pediatrician | 4,364 (5.3) | 7,527 | 58.0 |
| 12 | Internist | 14,634 (17.7) | 26,798 | 54.6 |
| 13 | Family physician/General practitioner | 19,967 (24.2) | 37,597 | 53.1 |
| 14 | Radiologist (incl. radiotherapist) | 863 (1.0) | 4,078 | 21.1 |
| 15 | Anesthetist | 601 (0.7) | 4,247 | 14.2 |
| 16 | Others | 3,599 (4.4) | 23,382^b,c^ | 15.4 |
| Total | | 82,511 (100.0) | 157,288 | 52.5% |

^a^If not other than [45].

^b^According to [45].

^c^Others (eg, psychotherapist, pathologist, pneumologist).
